# Characterization of lncRNA and mRNA profiles in rats with diabetic macroangiopathy

**DOI:** 10.1371/journal.pone.0243987

**Published:** 2020-12-30

**Authors:** Chan Yang, Ziyan Xie, Qiangfei Yang, Min Su, Ran Yan, Xueqin Cai, Xiaoxu Fu, Hong Gao, Lian Du, Wen Zhong, Chunguang Xie

**Affiliations:** 1 Hospital of Chengdu, University of Traditional Chinese Medicine, Chengdu, China; 2 Chengdu University of Traditional Chinese Medicine Clinical Medical College, Chengdu, China; 3 Department of Orthopaedics, The People’s Hospital of Jianyang city, Chengdu, China; 4 Chengdu University of Traditional Chinese Medicine, Chengdu, China; Université de Picardie Jules Verne, FRANCE

## Abstract

Diabetic macroangiopathy is part of the most common serious complications of diabetes. Previous studies indicate that lncRNAs involved in the process of diabetes and another vascular disease. However, their detailed mechanism of the lncRNAs involved in diabetic macroangiopathy has not been well characterized. In the present study, we generated rat models of diabetic macroangiopathy induced by High fat of 16weeks. A total of 15 GK rats were constructed as a test group, along with 15 Wistar rats set as control group, and thoracic aorta tissue from each group was collected. Whole genomic RNA sequencing was performed on thoracic aorta tissue; 3223 novel lncRNAs and 20367 annotated lncRNAs were indemnified in thoracic aorta samples, and 864 lncRNAs were expressed differently in the test and control groups. Gene ontology term enrichment showed the apparent enrichment of inflammatory response and cell apoptosis, which consistent with the results of H&E Staining, TUNEL Assay, and ELISA; Extensive literature reveals inflammatory response and cell apoptosis play an important role in the process of diabetic macroangiopathy. The results of the present study indicated that lncRNAs, especially Nrep. bSep08, Col5a1, aSep0, soygee.aSep08-unspliced, NONRATT013247.2, votar.aSep08-unspliced, etc, both participate in and mediate the process of inflammatory response, cell apoptosis. What’s more. Our research provides further insights into understanding of the basic molecular mechanisms underlying diabetic macroangiopathy.

## Introduction

Diabetic macroangiopathy is currently the disease with the highest rate of death and disability among the complications of diabetes [1]. The present study showed that disorders of glucose and lipid metabolism, inflammation, oxidative stress, insulin resistance, and apoptosis are closely related to the occurrence and development of diabetic macroangiopathy [2–5]. With the completion of the Human Genome Project, more and more studies have shown that long non-coding RNA (lncRNA) can participate in the regulation of gene expression at the level of epigenetic modification, transcription, and post-transcription [6]. Diabetes and its vascular disease are closely related with lncRNA [7]; However, their detailed mechanism of the lncRNAs involved in diabetic macroangiopathy was still in the enlightenment stage.

All experiments met all relevant regulatory standards. The male special pathogen-free (SPF) Wistar rats (total 15) and Goto-Kakizaki (GK) rats (total 15) from Chinese Academy of Sciences Shanghai Slack (SLAC) Laboratory Animals (Shanghai, China) weighing from 200 to 240g, were housed in an experimental animal center with an SPF-class experimental barrier system of Sichuan Academy of Traditional Chinese Medicine (SYXK (Chuan) 2018–100). The indoor temperature was maintained at 22 to 23 ° C and the humidity was maintained at 55% to 61%. The breeding method we adopted is divided into cages, 5 per cage, 6 cages in total. and the cages were sterilized with 20 pounds autoclave for 15 minutes daily. Besides, The indoor hygiene cleaning, cage changing/washing, etc, were all performed by professionally trained personnel. We turned on indoor fluorescent lights to simulate daylight from 8:00 to 20:00 and turned off the lights for the rest of the night to simulate night.

The GK rat model of diabetic macroangiopathy was generated through feeding with high fat. the constituents of the high-fat diet include 88.2% of common animal feed add 10% refined lard, 1.5% cholesterol, and 0.3% pig bile salt. While, the Wistar rats feeding in an ordinary diet, respectively 16 weeks. Finally, there were anesthetized by 20% Ulatan (1g/kg i.p) after no diet for 8 hours. We collected blood from the abdominal aorta which was centrifuged (3,000 rpm, 10 min) after being maintained at room temperature for 15 min. The serum was collected and stored at –20°C for ELISA. We peeled off the thoracic aorta immediately snap-frozen in liquid nitrogen and stored at –80°Cfor for further experiments.

## Materials and methods

This study was carried out in strict accordance with the recommendations in the Guide for the Care and Use of Laboratory Animals of the National Institutes of Health. The protocol was approved by the Committee on the Ethics of Affiliated Hospital of Chengdu University of Traditional Chinese Medicine. To ameliorate animals suffering, all related operations involving animals during the entire experiment were carried out by professional and skilled technicians. After the experiment, 20% Ulatan (1g/kg i.p) was used for rapid anesthesia.

Ulatran generally refers to Ethyl carbamate (C3H7NO2), 20% Uratan solution synthesis from 20g Ulatan and 0.9% NS 100ml mixed. The reason why we choose uratan as an anesthetic is that uratan has little effect on breathing.

### Animal model of diabetic macroangiopathy

#### Determination of FPG, H&E staining, TUNEL assay for apoptosis, and determination of IFN-γ, IL-4 in sera samples by ELISA

Every Saturday from 9 to 10 in the morning, the blood glucose meter tail vein blood sampling was utilized to test the fasting blood glucose of each group of rats. Before testing, fasting and water were not allowed for 8 hours. Blood was collected with a sterile disposable needle tail vein. The blood glucose meter quickly draws enough tail vein blood. After the value is shown, the blood glucose value is read and recorded truthfully.

The thoracic aorta was fixed in 10% formaldehyde for 48 h. The specimens were subjected to dehydration and permeabilization and then embedded in paraffin. The paraffin tissues were sliced into serial sections (0.4 mm) and stained with H&E. Pathological changes were observed under a microscope.

We used the TUNEL technique to detect apoptosis of thoracic aorta tissues. TUNEL apoptosis used the DeadEndTM Fluorescence TUNEL System (Promega, G3250), and was purchased from Promega Biotechnology (Beijing, China), and we performed the assay by the manufacturer’s instructions.

We detected IFN-γ, IL-4 in blood serum according to the instructions of the ELISA kit (Xitang, Shanghai, China). The concentration of each sample was calculated according to the optical density value and the standard curve.

#### RNA-Seq and bioinformatics analyses

**Fig 1:**
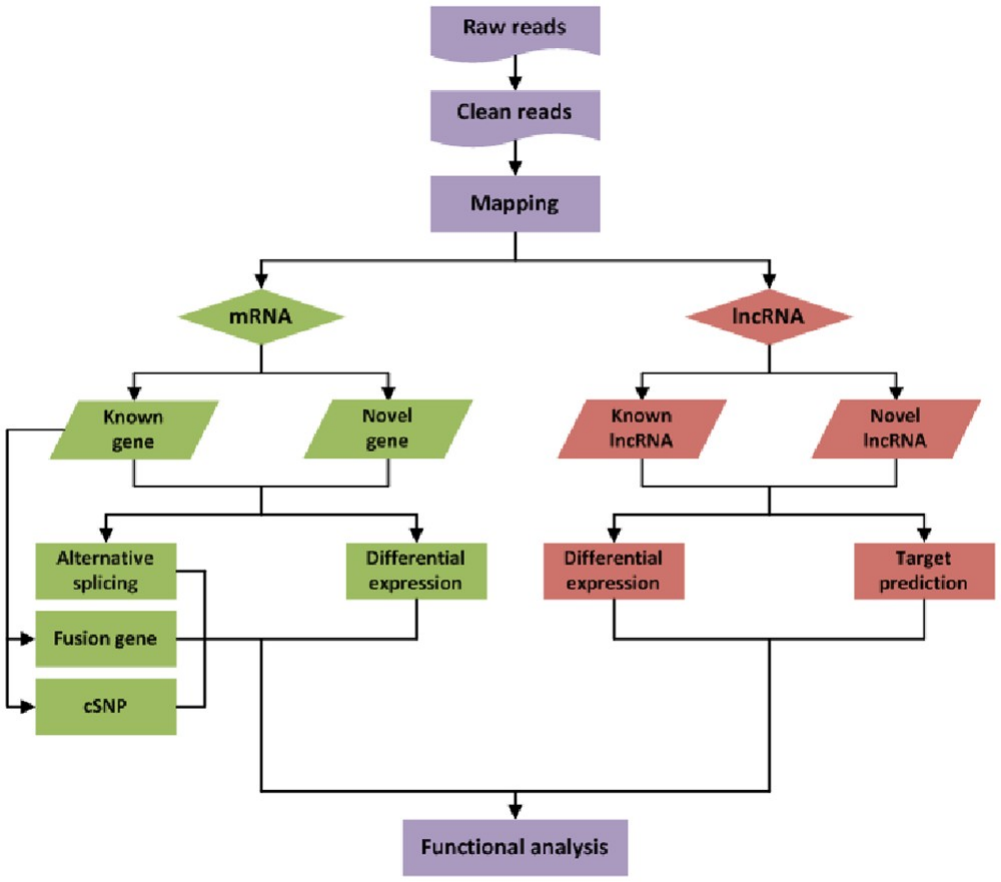
The procedures of RNA-seq and bioinformatics analyses.

Sequencing data were deposited with the NCBI. Total RNAs from rats subjected to experiment (n = 4) and controls (n = 4) were isolated and quality controlled. The preparation of whole-transcriptome libraries and next-generation sequencing were conducted by CHENGDU JUJIN BIOTECHNOLOGY CORPORATION (Chengdu, China). RNA-seq was performed on an Illumina Hiseq 4000 platform, and 150-bp-paired single-end reads were generated according to Illumina’s protocol. The procedures see Fig 1.

#### The constructed of mRNA—lncRNA networks

The differential lncRNA and differential mRNA co-expression was detected according to the Pearson correlation screen. The screening criteria were correlation coefficient coefficients> 0.7, P ≤ 0.05; Cytoscape software was used to generate the final differential lncRNA-differential mRNA co-expression network map.

### Statistical analysis

We analyzed the data via software of SPSS16.0 and the data were presented as the mean ± SEM. one-way analysis of variance was used, and the LSD test was used for pairwise comparison when the data conform to normal distribution and homogeneity of variance, otherwise, Using non-parametric tests (rank-sum test). If there is no special explanation, P <0.05 indicates statistical significance, that is, it was considered statistically significant.

## Results

### Model identification of diabetic macroangiopathy in GK Rat with high fat feeding

Our previous study shows that high-fat feeding can successfully induce macrovascular disease in GK rats. Thus, we generated a rat model of diabetic macroangiopathy induced via feeding of high fat, 16weeks. A total of 15 GK rats model was constructed and thoracic aorta from each was collected. The glucose levels >16.7 mmol/L was confirmed diabetic rat. The fasting plasma glucose (FPG) levels of each group list in Table 1. And the weight of rats in each group list in Table 2.

**Table 1:** FPG of rats in each group (X ± SEM, mmol/l), comparison of two groups.

Group	N	FPG
Control	15	6. 53±0.17
Model	15	22. 61±1. 54^★★^

^★★^*P*≤0.01.

**Table 2:** Weight of rats in each group (X ± SEM, g), comparison of two groups.

Group	N	Weight
Control	15	436. 2±11.0
Model	15	409. 7±6. 9^★^

^★★^*P*≤0.01,

^★^*P*≤0.05.

**Fig 2:**
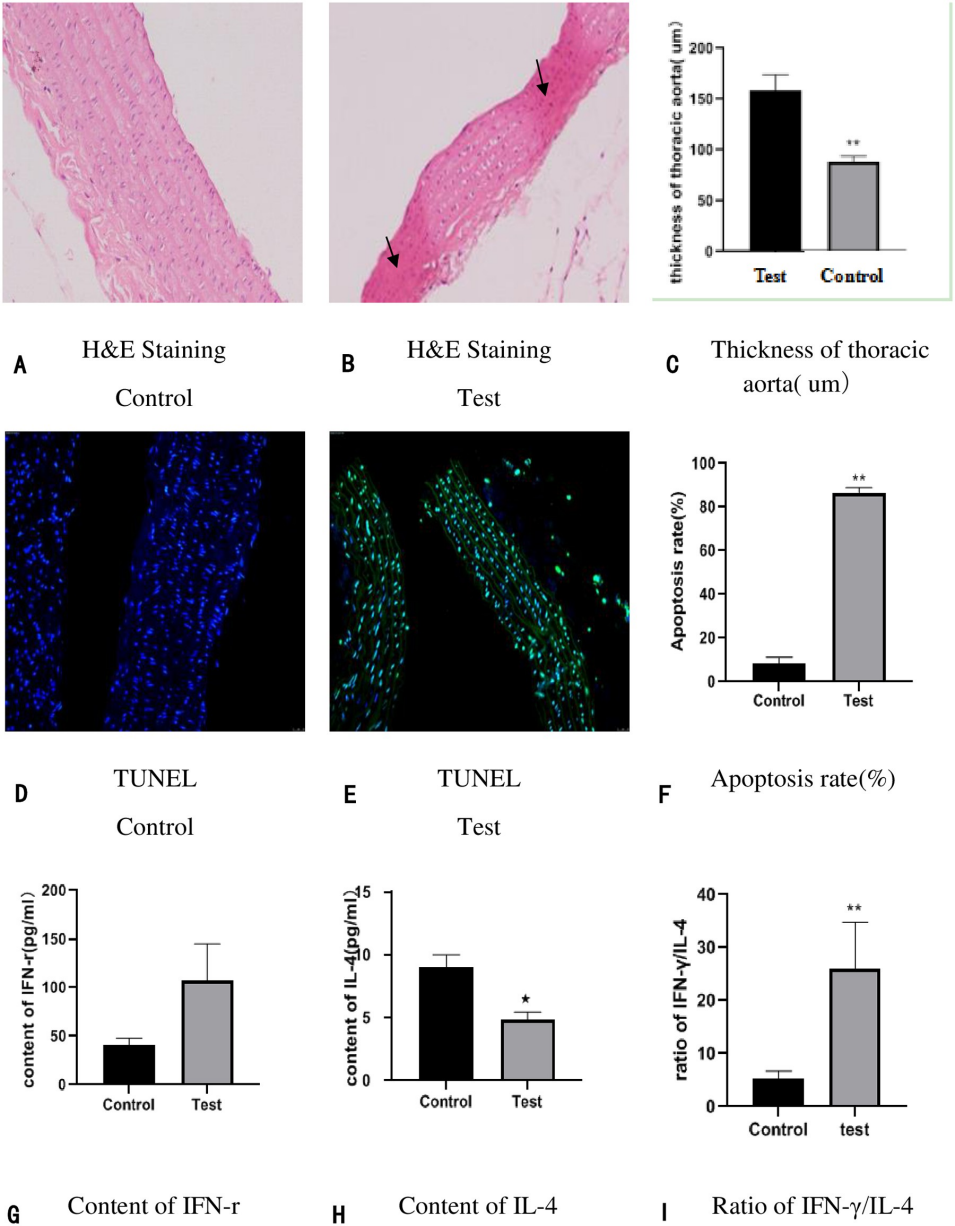
The basic characteristics between test group and the control group: (2A-2C): Pathological comparison in thoracic aorta tissue and vascular thickness through H&E staining (×200). n = 6 in each group. (2Dand 2F): Apoptosis detection in horacic aorta tissue by TUNEL staining (200×). n = 6 in each group. (2G-2H) Serum inflammatory factor (IFN-γand IL-4) were detected by ELISA; (2I): The ratio of IFN-γ/IL-4 were calculated via Spass, n = 15 in control and n = 14 in test group (1 GK rat died of ketosis caused by hyperglycemia); *p < 0.05 compared to the control; **p < 0.01 compared to the control.

In the control group, H&E staining showed that the inner cell membrane remains complete, and there is no swelling of endothelial cells, the arrangement of cells is normal (Fig 2A). While in the test group, H&E staining showed that the inner cell membrane was impaired, the endothelial cells swelled and shed, and there are disordered cells and the reduction of the cell middle membrane and the uneven thickness of the vascular wall (Fig 2B); Besides, The thickness of thoracic aorta was measured, and we found that the thickness of thoracic aorta was significantly decreased in the GK rats of diabetic macroangiopathy (Table 3, Fig 2C). Terminal deoxynucleotidyl transferase-mediated dUTP nick-end labeling (TUNEL) staining of thoracic aorta showed a statistically significant increase in apoptosis rates in GK rats (Fig 2D–2F). Besides, we examined serum inflammatory biomarkers (Interferon-γ (IFN-r) and interleukin-4 (IL-4), simultaneously calculated the ratio of IFN-γ/IL-4 (Fig 2F–2H); the results showed that the level of the IL-4 (Fig 2H) were significantly reduced and the ratio of IFN-γ/IL-4 (Fig 2I) were significantly raised in the GK rats of diabetic macroangiopathy. Regrettably, the content of IFN-r was no significantly raised in the test group (Fig 2G), we speculated that it may be related to the experiment time, no matter what, it still keeps rising.

**Table 3:** Thickness of thoracic aorta (um) ($\bar{\text{X}}\pm \text{S}$, um), comparison of two groups.

Group	N	12W
Control	6	158.23±15.47
Test	6	88.72±5. 26^★★^

^★★^*P*≤0.01,

^★^*P*≤0.05.

### RNA sequencing identifified the features of lncRNA and mRNA in diabetic macroangiopathy of GK rats

**Fig 3:**
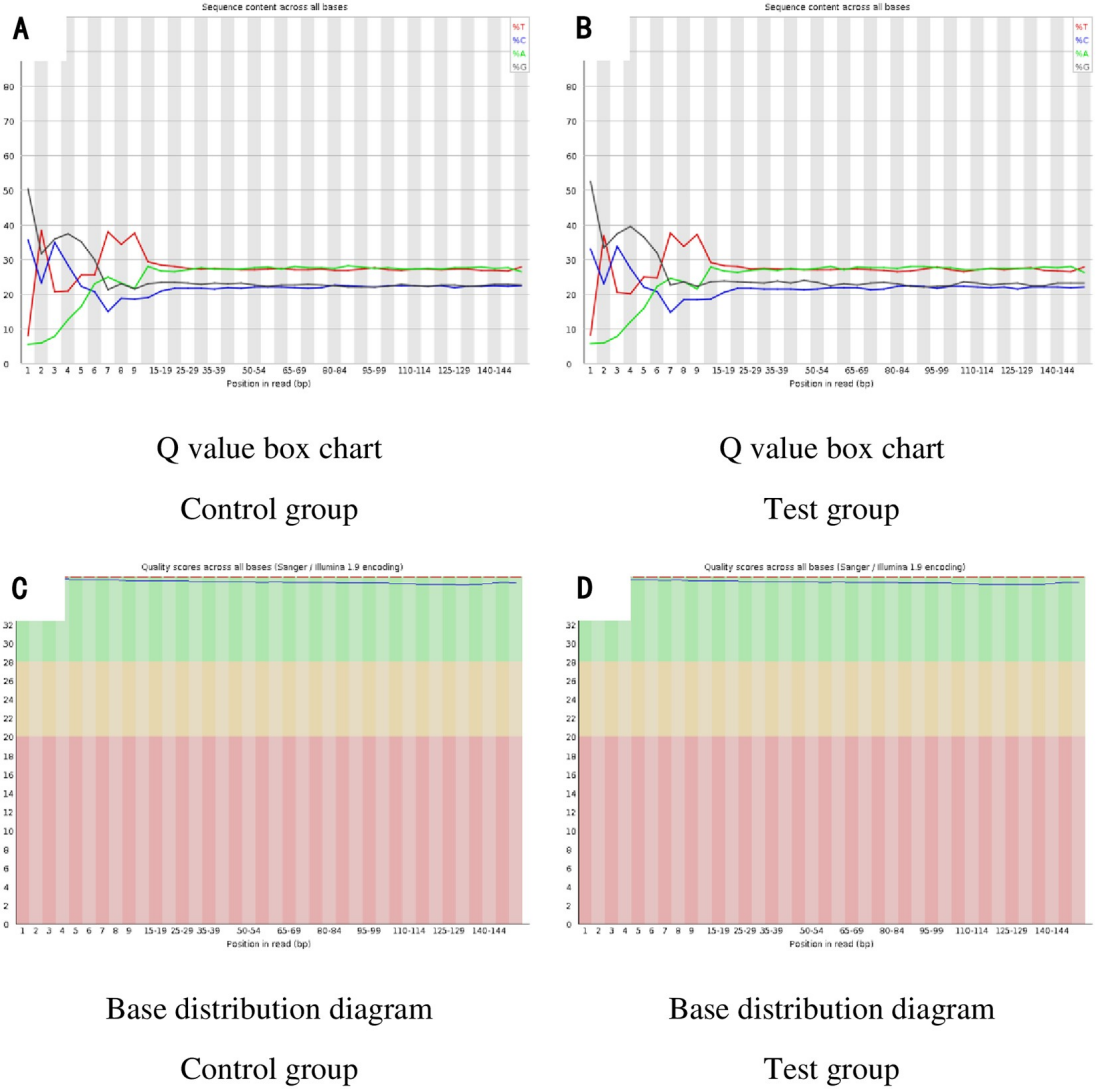
The basic information of data. (3A): Q value box chart of Control group; (3B): Q value box chart of Test group; (3C): Base distribution diagram of Control group; (3D): Base distribution diagram of Test group.

We analyzed RNA sequencing (RNA-seq) data from 4 rats randomly selected from each group thoracic aorta samples, in which 61,000,000–82,000,000 raw data and 57,000,000–79,000,000 clean data were obtained (Table 4). The classification of mapped reads in each thoracic aorta is shown in Table 5. The Fluorometer Quantitative Results (Table 6), Q value box chart (Fig 3A and 3B), and Base distribution diagram (Fig 3C and 3D) indicated all samples were sequenced per standards and data analysis was possible.

**Table 4:** The detailed information of RNA sequencing.

Sample ID	Raw reads	Clean reads	Clean ratio	rRNA trimed	rRNA ratio	No rRNA pair
Control 1	79,978,968	76,603,868	95.78%	76,431,593	0.22%	75,076,160
Control 2	61,334,798	57,848,275	94.32%	57,704,683	0.25%	56,410,154
Control 3	72,302,776	68,961,193	95.38%	68,866,487	0.14%	67,542,192
Control 4	82,377,968	79,307,418	96.27%	79,242,358	0.08%	78,125,230
Test 1	81,099,852	77,710,102	95.82%	77,510,349	0.26%	76,186,540
Test 2	75,341,414	72,013,132	95.58%	71,959,269	0.07%	70,642,008
Test 3	76,767,478	73,523,404	95.77%	73,449,426	0.10%	72,275,094
Test 4	79,208,782	75,857,586	95.77%	75,689,620	0.22%	74,322,602

Clean ratio = (Clean reads/Raw reads) %; rRNA ratio = [(Clean reads—rRNA trimed)/Clean reads] %.

**Table 5:** Results of mapping genome.

Samples_ID	All reads	Mapped reads	Mapped Pair Reads	Mapped broken-pair reads	Mapped Unique reads	Mapped Multi reads	Mapping ratio
Control 1	75,076,160	68,954,951	67,884,106	1,070,845	68,697,396	257,555	91.85%
Control 2	56,410,154	47,921,516	47,141,556	779,960	47,710,257	211,259	84.95%
Control 3	67,542,192	62,147,744	61,191,752	955,992	61,932,608	215,136	92.01%
Control 4	78,125,230	70,475,378	69,405,918	1,069,460	70,233,818	241,560	90.21%
Test 1	76,186,540	70,479,338	69,416,188	1,063,150	70,242,821	236,517	92.51%
Test 2	70,642,008	66,061,667	65,074,288	987,379	65,848,086	213,581	93.52%
Test 3	72,275,094	67,330,713	66,276,442	1,054,271	67,103,601	227,112	93.16%
Test 4	74,322,602	67,082,953	66,070,990	1,011,963	66,865,658	217,295	90.26%

Mapping ratio = Mapped reads/All reads, Mapped Unique reads.

**Table 6:** Qubit^®^ 2.0 fluorometer quantitative results.

No	Sample Name	Index No.	Con (ng/μL)	Peak Lenth (bp)
1	Control 1	V17	39.8	411
2	Control 2	V18	32.4	404
3	Control 3	V19	37.2	410
4	Control 4	V20	46.8	407
5	Test 1	V21	40.8	411
6	Test 2	V22	40.2	410
7	Test 3	V23	45.4	410
8	Test 4	V24	38.8	415

### Differential expression and cluster analysis of mRNAs and lncRNAs

We analyzed differential expression (DE) lncRNAs and mRNAs using significance analysis following the criterion q < 0.05 to prove whether lncRNAs were involved in the pathological process of diabetic macroangiopathy.

**Fig 4:**
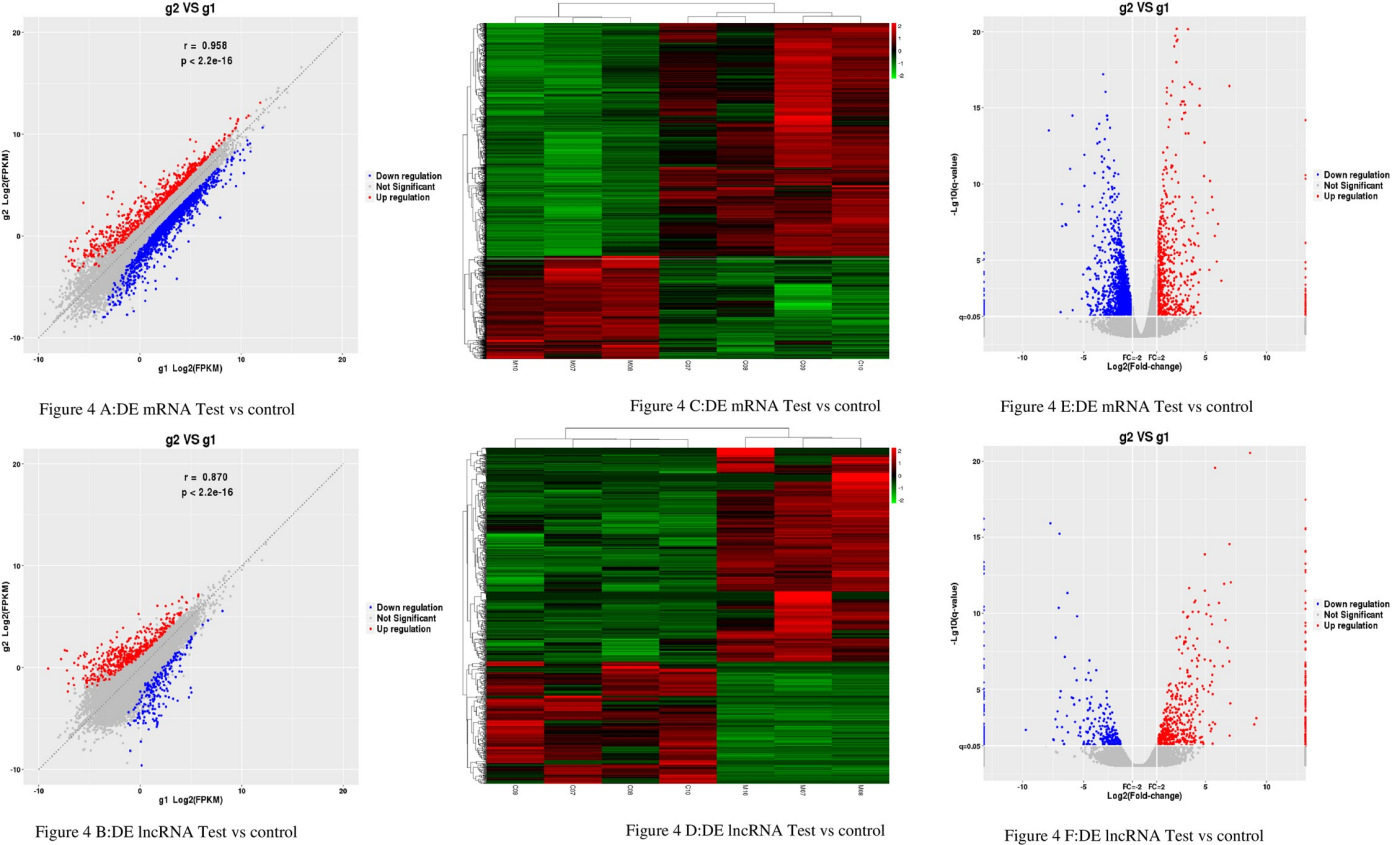
The Expression Profiling Changes of lncRNAs and mRNAs in Rats thoracic aorta Tissue: (4A and 4B) Volcano plot showing up-and down-regulated mRNAs (A) and lncRNAs (B) of rats thoracic aorta tissue in the test group compared with the control group; Red represents up-regulation, blue represents down-regulation, and gray represents no significant difference in gene expression. (4C and 4D) Heatmap of mRNAs (4C) and lncRNAs (4D) displaying hierarchical clustering of changed lncRNAs and mRNAs of rats thoracic aorta tissue in the test group compared with the control group; up- and downregulated genes are colored in red and blue, respectively. (4E and 4F) scatter plot showing up-and down-regulated mRNAs (E) and lncRNAs (F) of rats thoracic aorta tissue in the test group compared with the control group; Red represents up-regulation, blue represents down-regulation, and gray represents no significant difference in gene expression. g1 represents the control group while g2 represents the test group in all the figures.

The results showed that there were DE 2165mRNAs (664 upregulated and 1501 downregulated) and 864 DE lncRNAs (550 upregulated and 314 downregulated) (Fig 4A and 4B). The expression pattern of DE mRNAs and lncRNAs is shown using a clustered heatmap (Fig 4C and 4D).

The detailed information of the top 10 upregulated and 10 downregulated lncRNAs and mRNA summarized in Tables 7 and 8. These results reflect distinct lncRNAs and mRNA expression profiles between the test group and the control group, implying different underlying pathophysiology in diabetic macroangiopathy.

**Table 7:** The detailed information of the top 10 upregulated/downregulated mRNA.

Gene id	Gene name	Locus	log2fc	p	up/down
37436	Macc1	6	inf	6.34E-08	down
03136	Fcrla	13	inf	6.56E-08	down
24221	Mettl24	20	inf	1.92E-07	down
57008	AABR07010985.1	2	inf	1.87E-06	down
28576	Calml5	17	inf	2.76E-06	down
04341	LOC498222	13	inf	1.12E-05	down
29262	RGD1560017	3	inf	3.89E-05	down
55434	AABR07026641.1	16	inf	5.62E-05	down
20873	Nphs1	1	inf	0.000114	down
42468	AABR07066754.1	9	inf	0.000138	down
20985	Atp4a	1	inf	1.62E-17	up
13128	Tmem179	6	inf	1.21E-13	up
03284	Epn3	10	inf	2.20E-13	up
60029	AABR07072539.4	17	inf	1.08E-08	up
03273	Spata20	10	inf	3.39E-07	up
48522	LOC100909648	13	inf	9.92E-07	up
46250	LOC100911027	1	inf	4.98E-06	up
33983	Sohlh1	3	inf	1.85E-05	up
53562	Arx	X	inf	3.95E-05	up
47520	Rn60_5_0815.11	5	inf	5.94E-05	up

**Table 8:** The detailed information of the top 10 upregulated/downregulated lncRNAs.

lncRNA_id	lncRNA_name	length	locus	log2fc	P	up/down
NONRATT008127.2	RGD1559582.aSep08-unspliced	274	13	inf	8.37E-14	down
NONRATT026294.2	NONRATT026294.2	862	7	inf	3.69E-11	down
NONRATT007737.2	Rpl21.eSep08	601	12	inf	4.41E-10	down
NONRATT015712.2	NONRATT015712.2	2766	2	inf	1.01E-07	down
NONRATT023839.2	forwee.aSep08-unspliced	761	5	inf	3.93E-07	down
NONRATT016766.2	Gstm2.bSep08	402	2	inf	9.24E-06	down
NONRATT029734.2	LOC689856.aSep08-unspliced	538	9	inf	3.07E-05	down
NONRATT028171.2	Mobp.cSep08	791	8	inf	0.0001	down
NONRATT011382.2	Gtpbp3.cSep08	743	16	inf	0.0001	down
ENSRNOT00000078751	ENSRNOT00000078751	4140	2	inf	0.0002	down
NONRATT020147.2	NONRATT020147.2	611	4	inf	1.57E-26	up
NONRATT001894.2	toflo.aSep08	842	1	inf	2.00E-13	up
ENSRNOT00000085586	ENSRNOT00000085586	2426	17	inf	1.63E-06	up
NONRATT002575.2	Etfb.cSep08	614	1	inf	1.67E-06	up
NONRATT023574.2	Cap1.hSep08	552	5	inf	3.70E-06	up
NONRATT031178.2	NONRATT031178.2	622	X	inf	5.14E-05	up
NONRATT022004.2	korbo.aSep08-unspliced	325	5	inf	9.51E-05	up
NONRATT011770.2	peegar.aSep08-unspliced	1231	16	inf	0.0002	up
NONRATT025413.2	V-set.38.aSep08	411	6	inf	0.0006	up
NONRATT011006.2	speejar.aSep08-unspliced	911	14	inf	0.0014	up

#### Systematic functional analysis of differentially expressed lncRNAs and mRNAs

**Fig 5:**
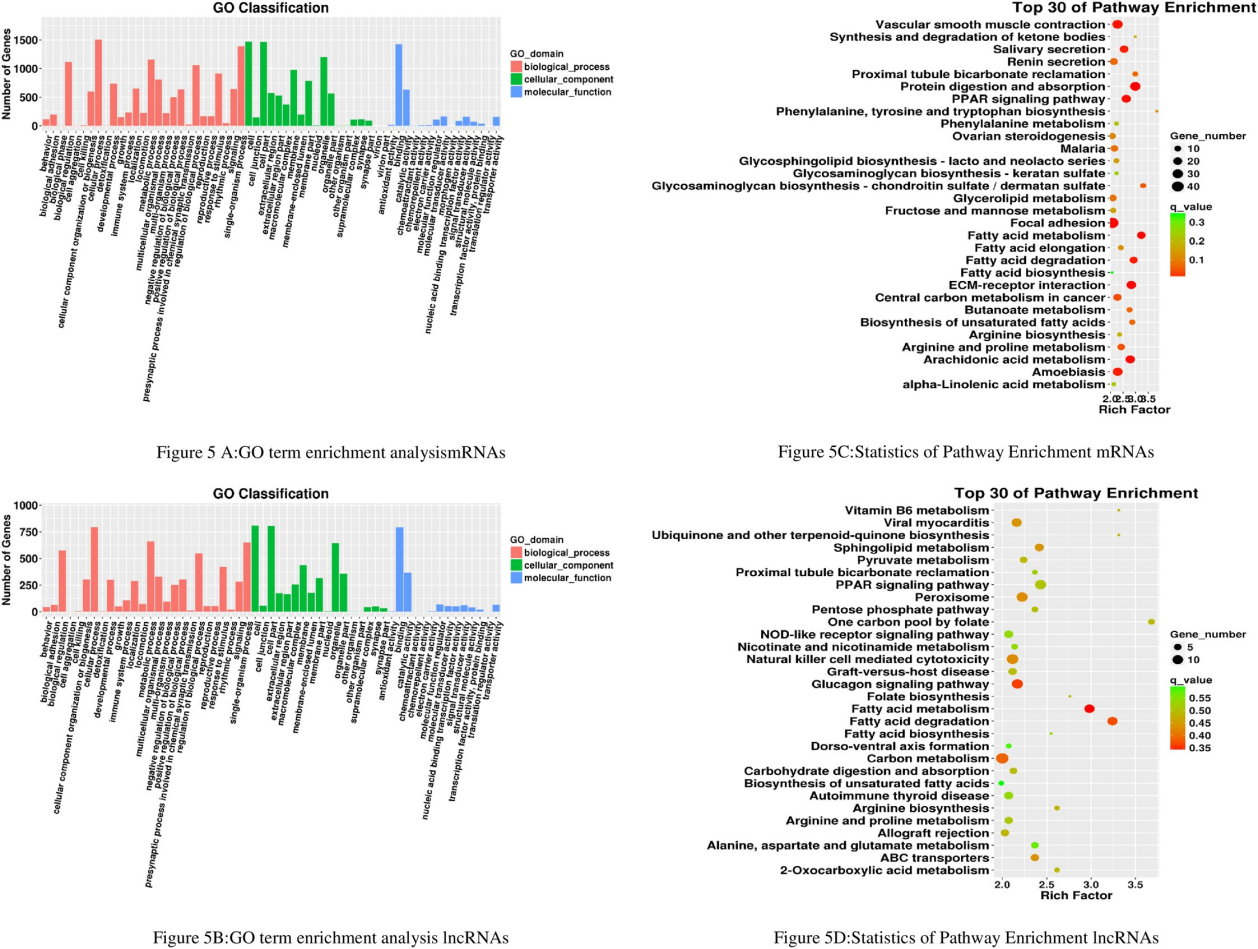
GO and KEGG Analyses of Differentially Expressed lncRNAs target Genes and mRNAs in rats thoracic aorta tissue from the test group and the control group: (5A) GO categories of differential mRNAs in rat heart tissue thoracic aorta tissue from the test group and the control group. (5B) GO categories of differential lncRNA s target genes in rat thoracic aorta tissue from the test group and the control group. (5C) KEGG analysis of differential mRNAs in rat thoracic aorta tissue from the test group and the control group. (5D) KEGG analysis of differential lncRNAs target genes in rat thoracic aorta tissue from the test group and the control group.

To clarify the possible functional significance of observed changes in lncRNAs and mRNAs levels between the test group and the control group, we performed a Gene Ontology (GO) term enrichment analysis. There were 5587 background genes in total. We summarized the significantly enriched GO terms of mRNAs (Fig 5A) and lncRNAs (Fig 5B) regarding the biological process, cellular component, and molecular function, respectively. Notably, we found that the DE of lncRNAs and mRNAs were similar and significantly related to inflammatory response and cell apoptosis in biological process term enrichment. Moreover, the GO term RNA regulation, metabolic processes, cell killing, cell aggregation, the cellular process, and immune systems was a significant enrichment on both the lncRNAs and mRNAs levels, which indicates that lncRNAs and mRNAs could make sense in the pathological process of diabetic macroangiopathy, and inflammatory response and cell apoptosis might play a key role in diabetic macroangiopathy. Apart from this, the enrichment of cellular components and molecular function also showed a similar pattern. Such as, cell junction, cell part, remembrance, and antioxidant activity were all enriched on the lncRNA and mRNA levels (Fig 5A and 5B). which revealed that complex pathological processes are involved in diabetic macroangiopathy.

To certificate if there were some specific pathways changed in diabetic macroangiopathy, we performed the Kyoto Encyclopedia of Genes and Genomes (KEGG) enrichment analysis in lncRNA target genes and mRNAs. It is noteworthy that the PPAR signaling pathway, fatty acid metabolism, and fatty acid degradation was also enriched in both lncRNAs and mRNAs (Fig 5C and 5D). The AMPK signaling pathway, JAK/STAT signaling pathway, Akt/mTOR pathway, Cellular Signaling Pathways were also enriched, which is consistent with previous research and consistent with the GO term enrichment results.

### Diabetic macroangiopathy-related differential lncRNA-mRNA co-expression network

**Fig 6:**
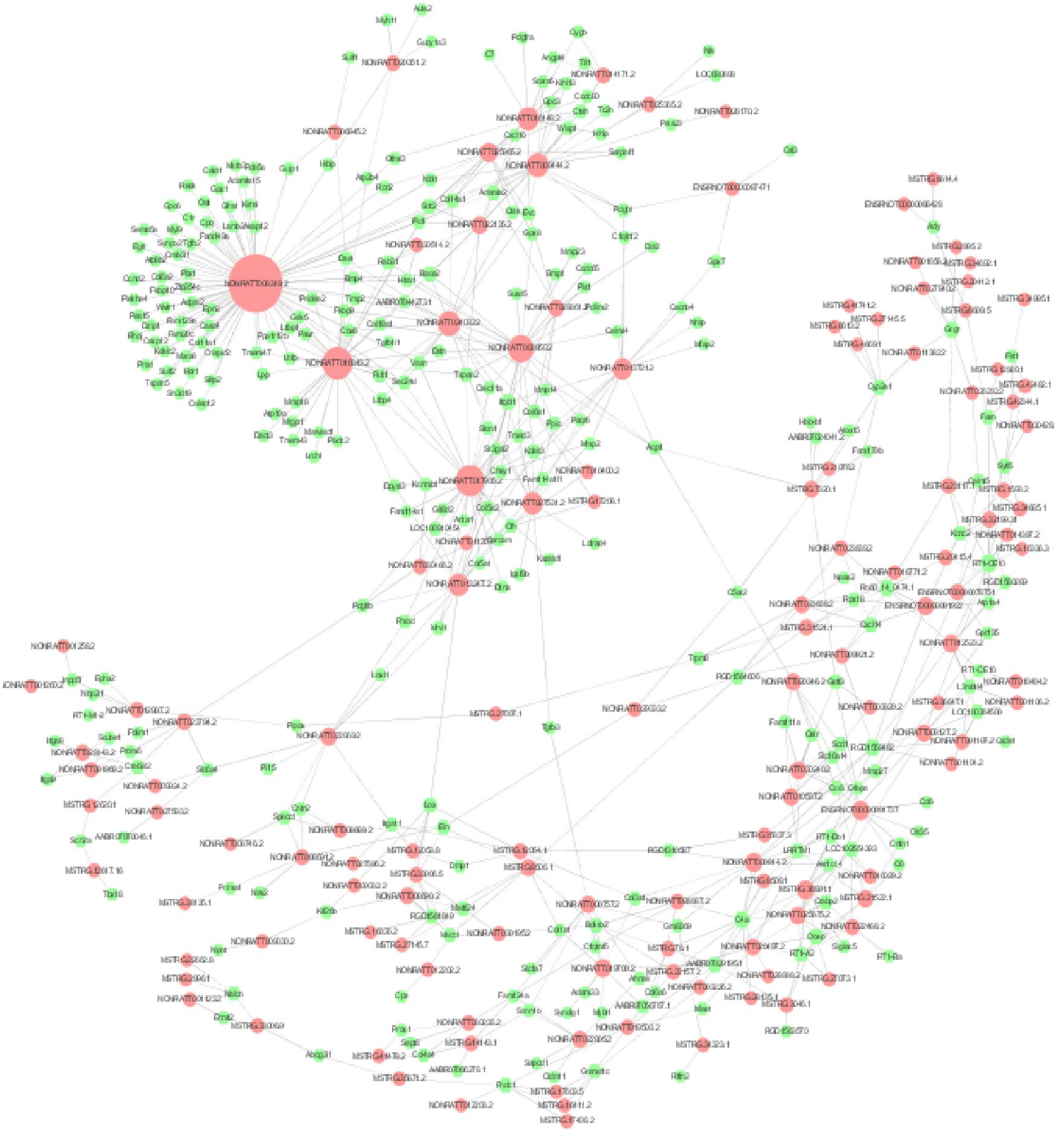
Diabetic macroangiopathy-related differential lncRNA-mRNA co-expression network: The red nodes represent lncRNAs and the green nodes represent mRNAs. The size of the nodes represents the weight of the gene in the network (degree). The larger the nodes, the larger the degree, and the more the number of differential mRNAs co-expressed around.

Pearson’s correlation coefficient (PCC) was computed between the expression values of each of the lncRNA-mRNA pairs across the two group’s samples (PCC>0.7, P≤0.05), respectively. Subsequently, the lncRNA-mRNA co-expression network was constructed by the Cytoscape (Fig 6). The core lncRNAs of the top 10 shown as follow in Table 9.

**Table 9:** The details of the top 10 lncRNAs (according to the degree).

lncRNA-id	LncRNA-name	dgree
NONRATT013721.2	Nrep.bSep08	883
NONRATT017935.2	Col5a1.aSep08	869
NONRATT011357.2	soygee.aSep08-unspliced	855
NONRATT013247.2	NONRATT013247.2	848
NONRATT024817.2	votar.aSep08-unspliced	845
ENSRNOT00000087471	ENSRNOT00000087471	836
NONRATT003950.2	zoklee.aSep08-unspliced	835
NONRATT029361.2	Col3a1.eSep08	833
MSTRG.17208.1	MSTRG.17208.1	825
NONRATT030466.2	glamu.aSep08-unspliced	825

## Discussion

Diabetic macroangiopathy, as a common complication of diabetes, usually causes multi-organ dysfunction and can lead to a high risk of disability and mortality [8]. Diabetic macroangiopathy, like a low-grade inflammation (chronic inflammation) state [9], is a direct result of the chronic low-grade inflammatory response of the blood vessel wall to endothelial cells. Various inflammatory mediators such as tumor necrosis factor, CRP, and IFN-γare closely related to the occurrence and development of diabetic macroangiopathy, Can be used as a predictor of diabetic macroangiopathy. Therefore, in this study, IL-4 and IFN-γ were selected as one of the markers for judging diabetic macrovascular injury. At the same time, the pathogenesis of diabetic macroangiopathy was discussed in depth from lncRNA.

To simulate the pathological process of diabetic macroangiopathy, we used a GK rat model fed with a high-fat of 16 weeks, which showed an obvious endothelial dysfunction (included endothelial injury and endothelial apoptosis). Many previous experiments have shown that the model is relatively stable and has a high recurrence rate. therefore, it is suitable for studying the mechanism of diabetic macroangiopathy [10].

At present, there are two types of gene sequencing including microarray sequencing and next-generation high-throughput sequencing. Compared with microarrays, next-generation sequencing has the advantages of high sensitivity, specificity, and the ability to discover important new lncRNAs. With the development of next-generation high-throughput sequencing technology, multiple lncRNAs have been found to play important roles in diabetes and its complications [5]. However, no related studies have been found to use whole-genome sequencing to report and compare changes in lncRNAs and mRNAs in diabetic macroangiopathy.

To recognize the basic characteristics of lncRNAs and mRNAs and find the potential role of lncRNAs in diabetic macroangiopathy, our study evaluated entire lncRNAs and mRNAs changing significantly in GK rat thoracic aorta tissues compared with the control group. What’s more, to increase the reliability and validity of sequencing, we exploited 4 GK rats of diabetic macroangiopathy compared with 4 control rats to find the ubiquitous differential expression lncRNAs and mRNAs. Base on quality control, this sequencing results had high reliability and quality. we predicted potential functions of lncRNAs and mRNAs by GO term enrichment and KEGG pathway enrichment, via bioinformatics analysis, and the results were beneficial for us to study the molecular mechanisms of diabetic macroangiopathy more clearly. Our study indicates that lncRNAs, including Nrep.bSep08, Col5a1.aSep0, soogee.aSep08-unspliced, NONRATT013247.2, votar.aSep08-unspliced, etc play key roles in the pathological process of diabetic macroangiopathy and provide a new direction toward understanding this pathological process.

We identified 864 DE lncRNAs between test group and control groups; the potential mechanisms of these lncRNAs are still unclear. We also conducted GO enrichment and found that the terms of biological processes occupied a great majority of the significantly enriched terms. Base on the KEGG enrichment results, we found that the PPAR signaling pathway, fatty acid metabolism, and fatty acid degradation were the most common pathways that emerged at both the lncRNA and mRNA levels. Even though some researches have shown the probable role of the aforementioned common pathways in some metabolic diseas [11–13], the regulatory relationship between lncRNAs and these pathways is still poorly understood, and more in-depth research is needed in the future basic studies.

With the introduction of the "Inflammatory Response Theory" by Professor Ross in 1993 [14], More and more studies have shown that diabetic macroangiopathy is an inflammatory disease [15]. It is believed that vascular endothelial injury is the initiating factor of diabetic macroangiopathy [16]. And is a direct result of the chronic low-grade inflammatory response of endothelial cells. Various inflammatory mediators such as IFN-γ and IL-4 are closely related to the occurrence and development of diabetic macroangiopathy [17]. Cell apoptosis, the pathological basis of endothelial dysfunction, will lead to the imbalance of the body’s homeostasis, and plays an important role in diabetic macroangiopathy [18]. We detected a significant difference in both cell apoptosis and the levels of the inflammatory factor, including IFN-γ, IL-4, and IFN-γ/IL-4 in thoracic aorta tissue from the test and control groups. These results are also matching with the results of the GO term and KEGG pathway enrichment.

Our study is to explore the role of LncRNAs in the pathological process of diabetic macroangiopathy. By applying whole-genome next-generation sequencing, we compared the differences of LncRNAs and mRNAs between test and control groups; this research indicates that LncRNAs play a critical role in the pathogenesis of diabetic macroangiopathy. and this provided a new perspective for the study of the pathological mechanism of diabetic macroangiopathy, and also provided new ideas for the future diagnosis and treatment of diabetic macroangiopathy.

Problems and Prospects: This experiment explored the mechanism of mRNA/lncRNA expression profile differences, and also screened out related differential genes, but no relevant verification was performed. The next step could be PCR quantification, WB protein qualitative and quantitative verification, and methyl In chemical verification, primary cell culture, siRNA interference, and gene knockout can also be used to verify related core lncRNA.
